# Increased expression of ECT2 predicts the poor prognosis of breast cancer patients

**DOI:** 10.1186/s40164-022-00361-3

**Published:** 2022-12-26

**Authors:** Ming Yi, Di Zhang, Bin Song, Bin Zhao, Mengke Niu, Yuze Wu, Zhijun Dai, Kongming Wu

**Affiliations:** 1 Department of Breast Surgery, The First Affiliated Hospital, College of Medicine, Zhejiang University, Hangzhou, 310000 China; 2 Cancer Center, Shanxi Bethune Hospital, Shanxi Academy of Medical Science, Tongji Shanxi Hospital, Third Hospital of Shanxi Medical University, Taiyuan, China; 3 Department of Oncology, Tongji Hospital of Tongji Medical College, Huazhong University of Science and Technology, Wuhan, 430030 China

**Keywords:** ECT2, Breast cancer, Predictive biomarker, Rho GTPases, Prognostic factor

## Abstract

Breast cancer is the most common malignancy and the second leading cause of cancer-related death in women. Recent studies have indicated that aberrant activation of Rho GTPases relates to the malignant properties of breast cancer cells. As the guanine nucleotide exchange factor of Rho GTPases, the role of ECT2 (epithelial cell transforming 2) in breast cancer is still unclear. Tissue microarrays and multiple public databases were utilized to investigate the relationship between ECT2 level and clinical-pathological features of breast cancer patients. Kaplan Meier-plotter online tool and tissue microarray with survival information were used to investigate the predictive value for breast cancer. Here, we found increased ECT2 level was highly associated with advanced TNM stage, poor differentiation, and loss of hormone receptors of breast cancer. Gene expression profile showed that ECT2 level was closely correlated to cell-proliferation-associated pathways. Integration analysis using public databases and tissue microarray indicated that high ECT2 was an adverse prognostic factor for breast cancer patients. We believe the ECT2 level might be a valuable complement for commercially available predictors such as the 21 genes test. Furthermore, ECT2 would be a novel target for drug development for breast cancer.

**Supplementary Information:**

The online version contains supplementary material available at 10.1186/s40164-022-00361-3.

## Introduction

Breast cancer is the most common malignancy and the second leading cause of cancer-related death in women [1–4]. According to the level of estrogen receptor (ER), progesterone receptor (PR), and human epidermal growth factor 2 (Her2), breast cancers are classified into multiple subtypes: Luminal, Her2 overexpression, Basal-like, and Normal-like [5]. Propelled by the fundamental investigation of tumor biology, breast cancer therapeutics have progressed substantially in the past decades [6]. Locoregional tumor load, together with molecular subtype, determines the therapeutic regimen of breast cancer [7–10]. Further exploration of molecular mechanisms contributing to tumor progression is meaningful to the development of novel therapies [11–15]. Notably, immunotherapy, especially immune checkpoint inhibitors, brings new hope to the treatment of breast cancer [16–20]. Recent studies have indicated that aberrant activation of Rho GTPases relates to the malignant biological behavior of breast cancer cells [21–23].

ECT2 (epithelial cell transforming 2) is a guanine nucleotide exchange factor of Rho GTPases (e.g. RhoA, Rac1, and Cdc42) [24]. ECT2 converts the status of Rho GTPases from inactive state to active state via replacing bound GDP with GTP, further promoting actin remodeling [25]. It has been verified that ECT2 mainly plays a vital role in cytokinesis. In M phase, ECT2 is recruited to central spindle and activates Rho signaling pathway, subsequently inducing contractile ring formation and contraction [26]. Loss of ECT2 interferes with cytokinesis without remarkable effects on mitosis, which ultimately promotes the generation of binucleate cells [27].

Besides normal cellular activity, ECT2 also participates in malignant transformation, tumor initiation, and metastasis [28]. The tumorigenic role of ECT2 changes along with its subcellular location. Full-length ECT2 protein contains a nuclear localization signal (N-terminal) and dominantly distributes in the nucleus [29]. Independent of cytokinesis regulation, nuclear ECT2 promotes malignant transformation in other manners [30]. In lung adenocarcinoma cells, nuclear ECT2 recruits and activates Rac1, which increases ribosome biogenesis and supports transformed growth (anchorage-independent growth) [31, 32]. Besides, in ovarian cancer cells, ECT2 is retained in cytoplasm by binding to protein kinase Cι-Par6α complex [30]. Cytoplasmic ECT2-protein kinase Cι-Par6α complex subsequently activates Rac1-Pak-Mek-Erk signaling pathway, which promotes cell cycle progression and proliferation [30]. Moreover, explorations in human hepatocellular carcinoma cell confirmed ECT2 as a multiple-functional oncogenic protein that upregulates Rho-Erk signal, promotes cell proliferation, suppresses apoptosis, and induces distant metastasis [33]. It has been detected that ECT2 was overexpressed in various cancers, including non-small lung cell cancer, glioblastomas, and prostate cancer [31, 34–36]. Generally, high ECT2 level is an unfavorable factor for patient prognosis [37, 38]. However, there are rare studies investigating the role of ECT2 in breast cancer.

In this study, by combining samples from tissue microarrays and gene expression data from public databases, we retrospectively evaluated the expression of ECT2 in breast cancer and non-cancer tissues. Moreover, we explored the relationship between ECT2 abundance and clinic-pathologic features as well as clinical outcomes of patients with breast cancer. Collectively, we explored the involvement of ECT2 in breast cancer development which would be a potential predictive biomarker and treatment target.

## Materials and methods

### Breast tissue samples

Tissue microarray Br2082a was obtained from Xi’an Alena-bio Ltd which contained 32 metastatic carcinoma, 120 primary carcinoma, 8 fibroadenoma, 16 hyperplasia, 16 inflammation, and 16 adjacent normal breast tissues. However, some clinical-pathological parameters and surviving data of samples were not given in Br2082a. Therefore, another tissue microarray with detailed pathological parameters (HBreD145Su01, Shanghai Outdo Biotech Ltd.) was involved for further analysis. The clinical-pathological features of breast cancer patients in HBreD145Su01 cohorts are shown in Table 1.

**Table 1:** Correlations between ECT2 expression and clinic-pathological features of 120 breast cancer patients in immunohistochemistry chip (HBreD145Su01)

Variables	N	ECT2 expression		P value
		< 8 (low expression)	≥ 8 (high expression)	
Age				0.224^a^
< Median(51)	57	28	29	
≥ Median (51)	63	24	39	
Tumor location				0.961^a^
Left	58	25	33	
Right	62	27	35	
Grade				0.073^a^
Grade 1	25	16	9	
Grade 2	90	44	56	
Stage				0.043^a^
Stage I–II	75	37	38	
Stage III–IV	43	13	30	
Subtype				0.0041^a^
Luminal	89	46	43	
Her2 enriched	13	4	9	
Basal	18	2	16	

^a^Pearson Chi-square test

### Public breast cancer datasets acquisition and process

We searched GEO database (http://www.ncbi.nlm.nih.gov/geo/) to select eligible breast cancer datasets for pooled analysis. The searching and selecting strategies followed the methods we previously described [39]. The detailed information of involved datasets in the meta-analysis was listed in Additional file 1: Table S1. Odds ratio (OR) with 95% confidence interval (95% CI) was utilized to assess the correlation between *ECT2* mRNA level and clinic-pathological markers. Patient outcomes, including overall survival (OS), relapse-free survival (RFS) and metastasis-free survival (MFS) were evaluated by hazard ratio (HR) and 95% CI. The Stata software (version 12.0) was used in this meta-analysis. Transcriptional profiling of *ECT2* gene and other genes was downloaded from TCGA database (https://xenabrowser.net/), processed and analyzed by SPSS software (version 24.0).

### Online analysis tool

Online analysis tool Kaplan Meier-plotter could plot Kaplan–Meier survival curve with log-rank test analysis (http://kmplot.com). The background data of Kaplan Meier-plotter are obtained from GEO and TCGA databases. Adopting median *ECT2* expression as cutoff value, Kaplan–Meier survival curves were generated and downloaded from this website.

### Immumohistochemical staining assay

Immunohistochemical (IHC) staining was conducted following the standard protocol described previously [40]. The specific primary antibody against ECT2 (catalog no. 07-1364; Millipore Corporation, Billerica, MA, USA, 1:150) was used for the IHC assay. The stained images were captured by a light microscope with the image processing system (Sunny Optical, China). The IHC scores were evaluated by two experienced pathologists without patient information. Based on Fromowitz Standard, the multiplication of intensity and proportion of positively stained cancer cells represents the abundance of ECT2. The intensity was scored according to color shade: 0 (no staining), 1 (light yellow), 2 (yellow–brown), and 3 (brown). The proportion of stained cancer cells was scored as: 1 (0–25%), 2 (26–50%), 3 (51–75%), and 4 (76–100%).

### Bioinformatics analysis

The biological significance of ECT2 for breast cancer was explored using bioinformatics analysis. Based on TCGA and GSE25066 datasets, the enriched Gene ontology (GO) terms and Kyoto Encyclopedia of Genes and Genomes (KEGG) pathways in high ECT2 tumors were calculated. Pathways or Terms with adjusted P value < 0.05 and fold change > 2 were regarded as statistically significant [18, 41]. Gene Set Enrichment Analysis (GSEA) was performed to explore the enriched pathways in high ECT2 tumors [19, 42]. R software (4.1.2) and packages DESeq2, ggplot2, and ClusterProfile were used in this assay.

### Statistical analysis

The Student's t-test was used to compare the difference between two groups with the significance cutoff as 0.05. Correlations were analyzed using Person χ^2^ test. The cumulative survival was analyzed using Kaplan–Meier curve with log-rank test. Univariate and multivariate analyses were conducted by the Cox proportional hazards regression model.

## Results

### Increased ECT2 expression in breast cancer

**Fig. 1:**
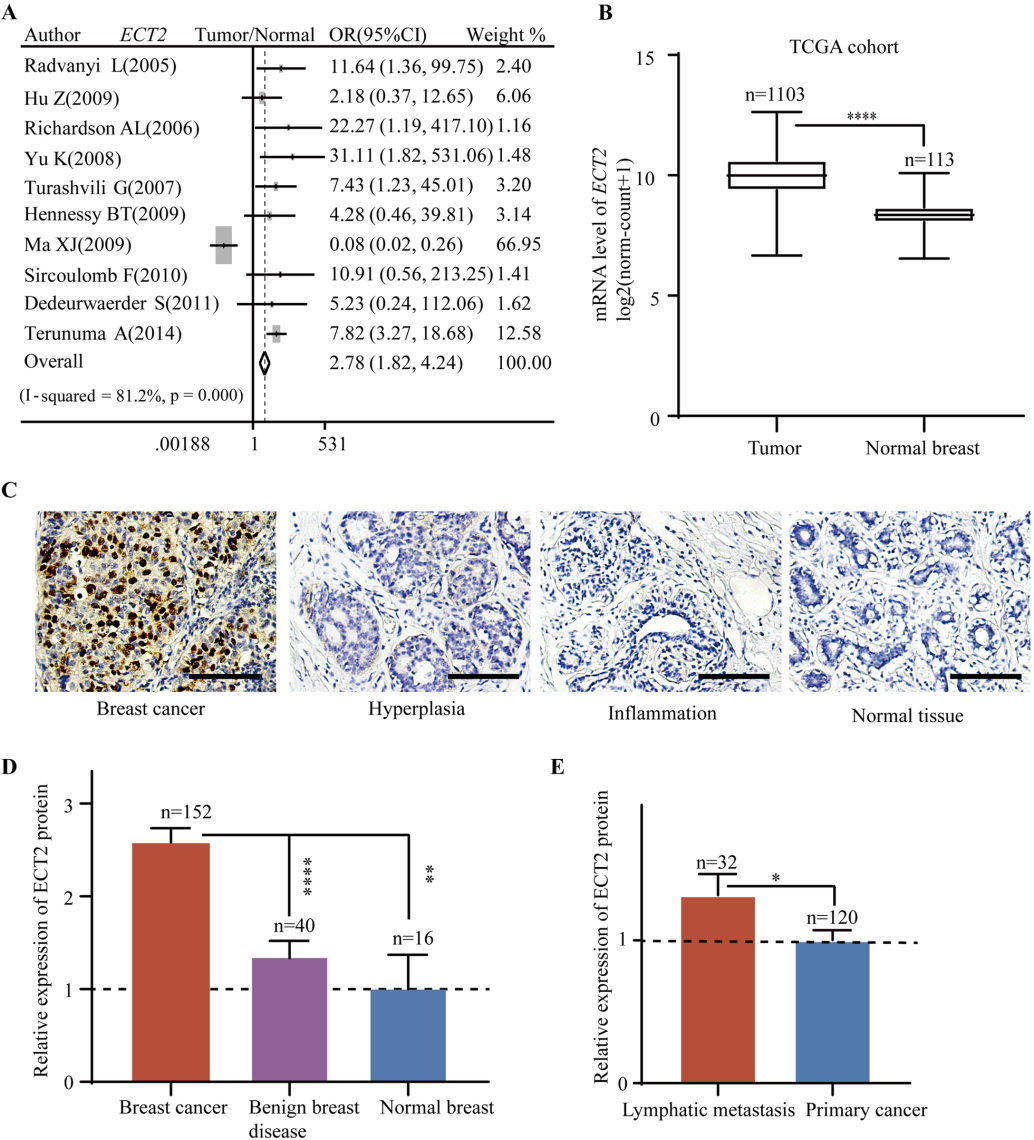
The expression level of ECT2 in breast cancer, benign disease, and normal breast tissues.** A** Pooled analysis of GEO datasets showing *ECT2* mRNA level in breast cancer and normal tissues. **B** TCGA breast cancer dataset showing the level of *ECT2* mRNA level in breast cancer and normal tissues. **C** The representative immunohistochemical staining images showing ECT2 protein expression in breast cancer and normal tissues. **D** Histogram showing ECT2 protein expression in breast cancer, benign disease, and normal breast tissues. **E** Histogram showing ECT2 protein expression in lymphatic metastasis and primary breast cancer tissues. Scale bar: 100 μm

To explore the role of ECT2 in breast cancer, we first assessed the *ECT2* expression in breast cancers and normal breast tissues. Pooled analysis of GEO datasets demonstrated that *ECT2* mRNA level was upregulated in breast cancer tissues relative to normal tissues (OR = 2.78, 95%CI = 1.82–4.24) (Fig. 1A). *ECT2* mRNA data from TCGA showed significantly elevated *ECT2* mRNA in breast cancer tissues as well (P < 0.0001) (Fig. 1B). Breast samples of the tissue microarray contained breast cancer, benign disease, and normal breast tissues. IHC results indicated that ECT2 protein was increased in breast cancer tissues compared with non-cancer tissues (Fig. 1C, D). Among 152 breast cancer tissues, samples from lymphatic metastasis exhibited higher ECT2 expression relative to primary breast cancer (Fig. 1E).

### Correlations between ECT2 expression and clinic-pathologic features of breast cancer patients

In order to further verify the clinical significance of increased ECT2 for breast cancer patients, we interrogated the relationship between ECT2 expression and clinic-pathologic parameters, including TNM stage (based on tumor size, lymph node involvement and distant metastasis), and molecular subtype.

**Fig. 2:**
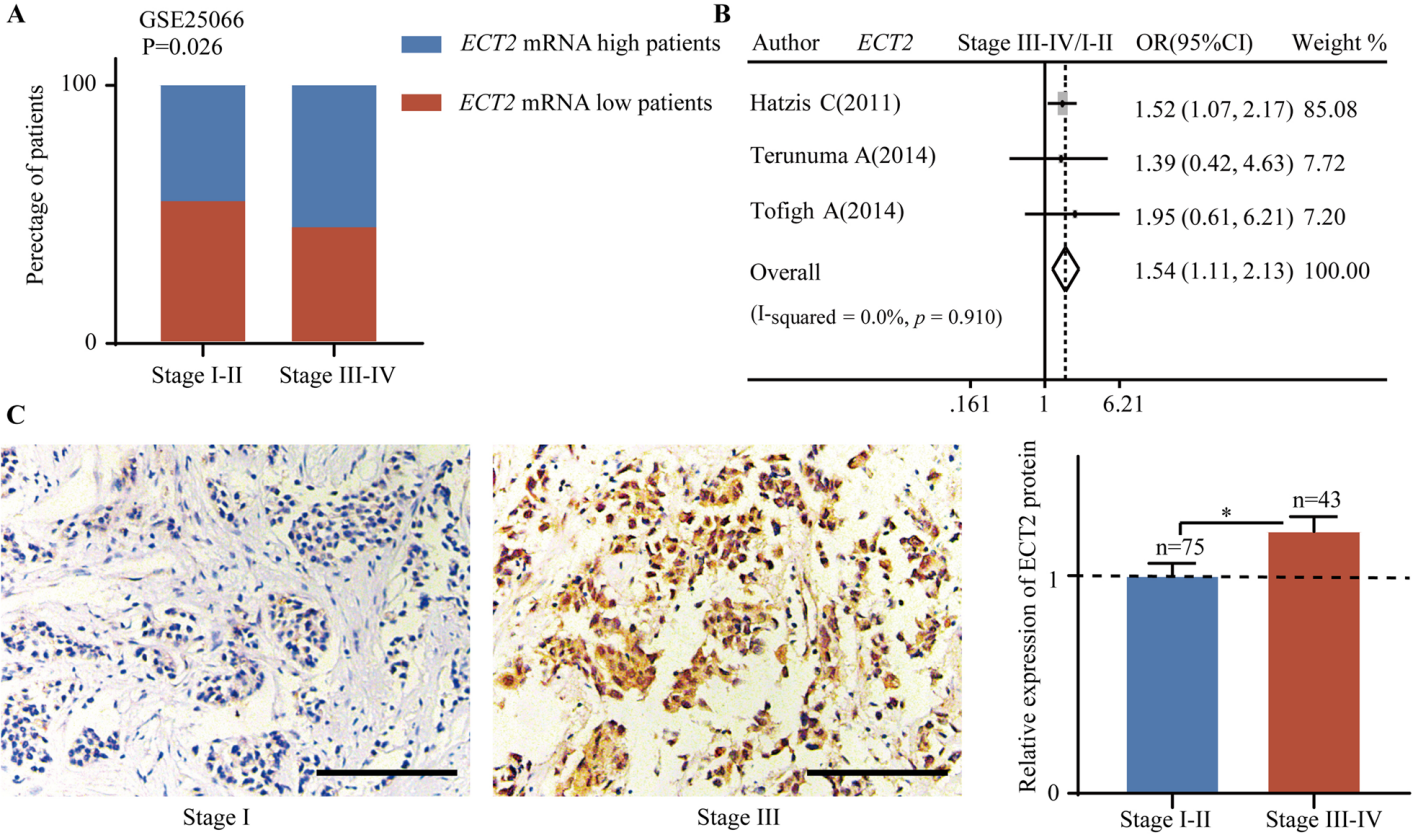
The correlation between ECT2 expression and TNM stage. **A** Gene expression profile from GSE25066 showing that the percentage of patients with high *ECT2* mRNA and low high *ECT2* mRNA in different TNM stage groups. **B** Pooled analysis showing the correlation between *ECT2* mRNA and TNM stage. **C** Data from tissue microarray showing the relationship between ECT2 protein level and TNM stage. Scale bar: 100 μm

ECT2 expression was related to TNM stage. Gene expression profile from GSE25066 showed that the percentage of patients with upregulated *ECT2* mRNA was higher in the stage III–IV group (Fig. 2A). Pooled analysis showed that increased *ECT2* mRNA significantly correlated with advanced TNM stage (Pooled OR = 1.54, 95%CI 1.11–2.13) (Fig. 2B). ECT2 protein abundance data from tissue microarray demonstrated that upregulated ECT2 protein correlated with advanced TNM stage (P < 0.05) (Fig. 2C).

**Fig. 3:**
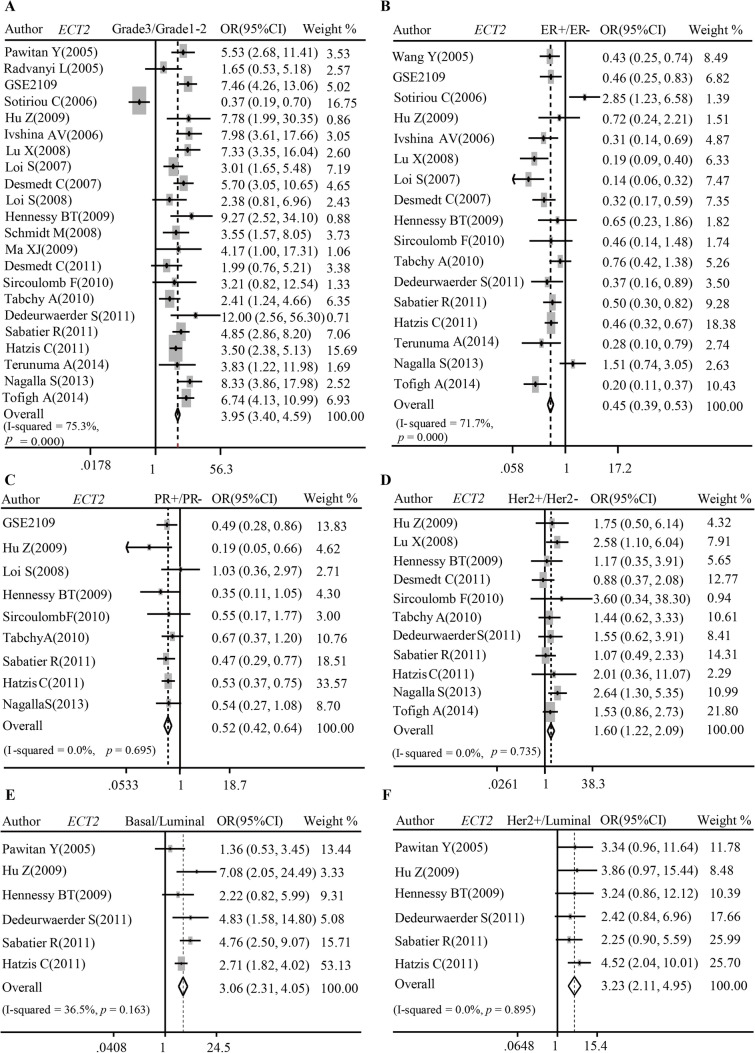
Pooled analysis showing the correlations between *ECT2* mRNA and molecular biomarkers of breast cancer. **A** Pooled analysis showing the correlations between *ECT2* mRNA level and differentiation grade. **B** Pooled analysis showing the correlations between *ECT2* mRNA level and ER status. **C** Pooled analysis showing the correlations between *ECT2* mRNA level and PR status. **D** Pooled analysis showing the correlations between *ECT2* mRNA level and HER2 status. **E**, **F** Pooled analysis showing the correlations between *ECT2* mRNA level and subtypes of breast cancers

**Fig. 4:**
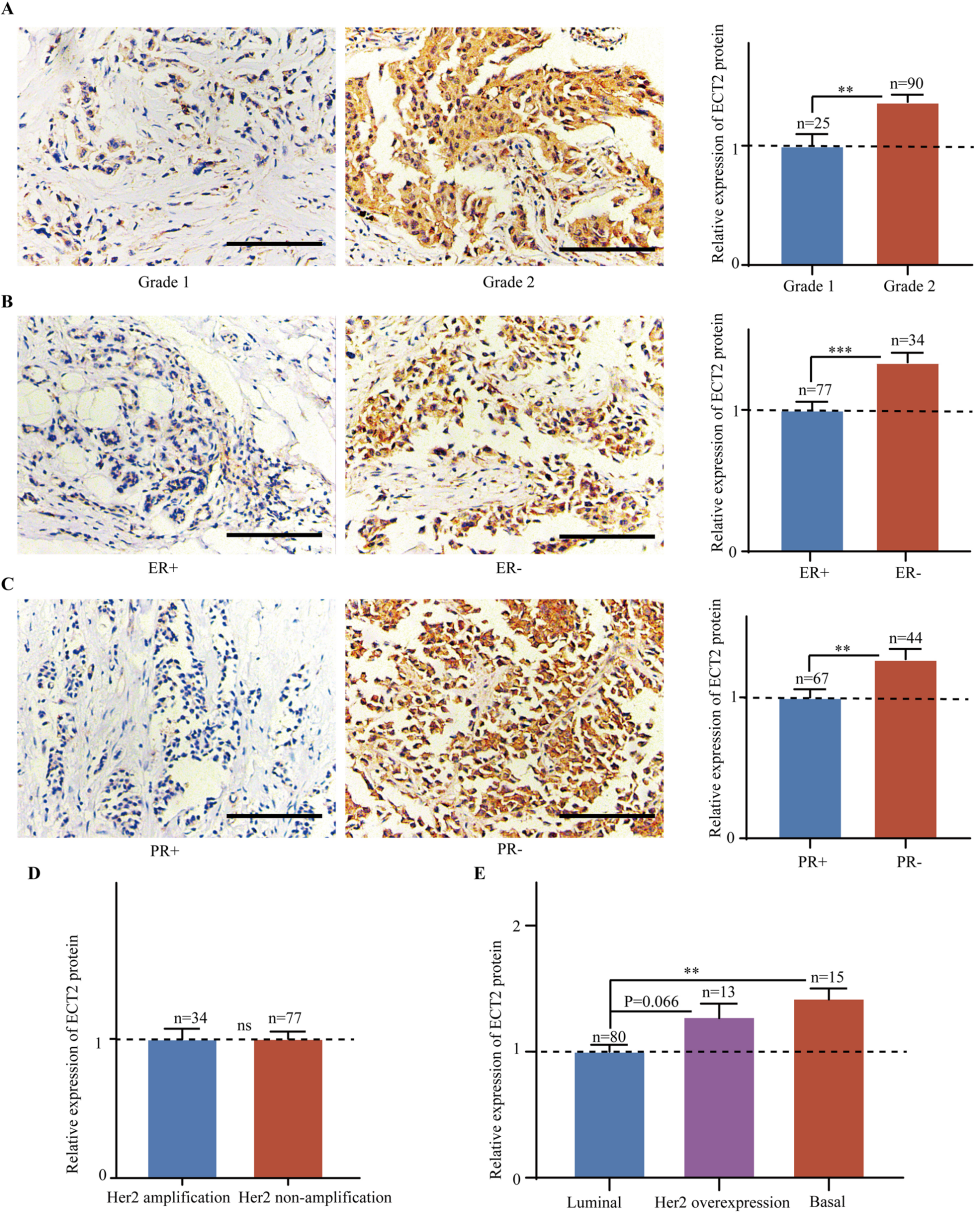
Data from tissue microarray showing the relationship between ECT2 protein level and molecular biomarkers of breast cancer. **A** The representative immunohistochemical staining images showing ECT2 protein expression in Grade 1 and Grade 2 breast cancers. **B** The representative immunohistochemical staining images showing ECT2 protein expression in ER+ and ER− breast cancers. **C** The representative immunohistochemical staining images showing ECT2 protein expression in PR+ and PR− breast cancers. **D** Histogram showing ECT2 protein expression in Her2-amplificated and non-amplificated breast cancers. **E** Histogram showing ECT2 protein expression in different subtypes of breast cancers. Scale bar: 100 μm

Elevated ECT2 expression was correlated to molecular biomarkers and subtypes of breast cancer. ECT2 manifested remarkably high transcriptional level in poorly differentiated tumor (Pooled OR = 3.95, 95%CI 3.40–4.59) (Fig. 3A) but low transcriptional level in ER + (Pooled OR = 0.45, 95%CI 0.39–0.53) (Fig. 3B) and PR + (Pooled OR = 0.52, 95%CI 0.42–0.64) patients (Fig. 3C). On the contrary, *ECT2* mRNA was upregulated in Her2-overexpressed tissue (Pooled OR = 1.60, 95%CI 1.22–2.09) (Fig. 3D), Basal-like subtype (Pooled OR = 3.06, 95%CI 2.31–4.05) (Fig. 3E), as well as Her2 overexpression subtype (Pooled OR = 3.23, 95%CI 2.11–4.95) (Fig. 3F). IHC scoring results showed that the abundance of ECT2 protein was markedly upregulated in poorly differentiated (Fig. 4A), ER- (Fig. 4B), PR- (Fig. 4C) breast cancers. However, there was no significant difference between Her2-amplificated and non-amplificated cancers (Fig. 4D). Additionally, ECT2 protein level was highest in Basal-like subtype breast cancers (Fig. 4E).

### The biological significance of ECT2 for breast cancer

**Fig. 5:**
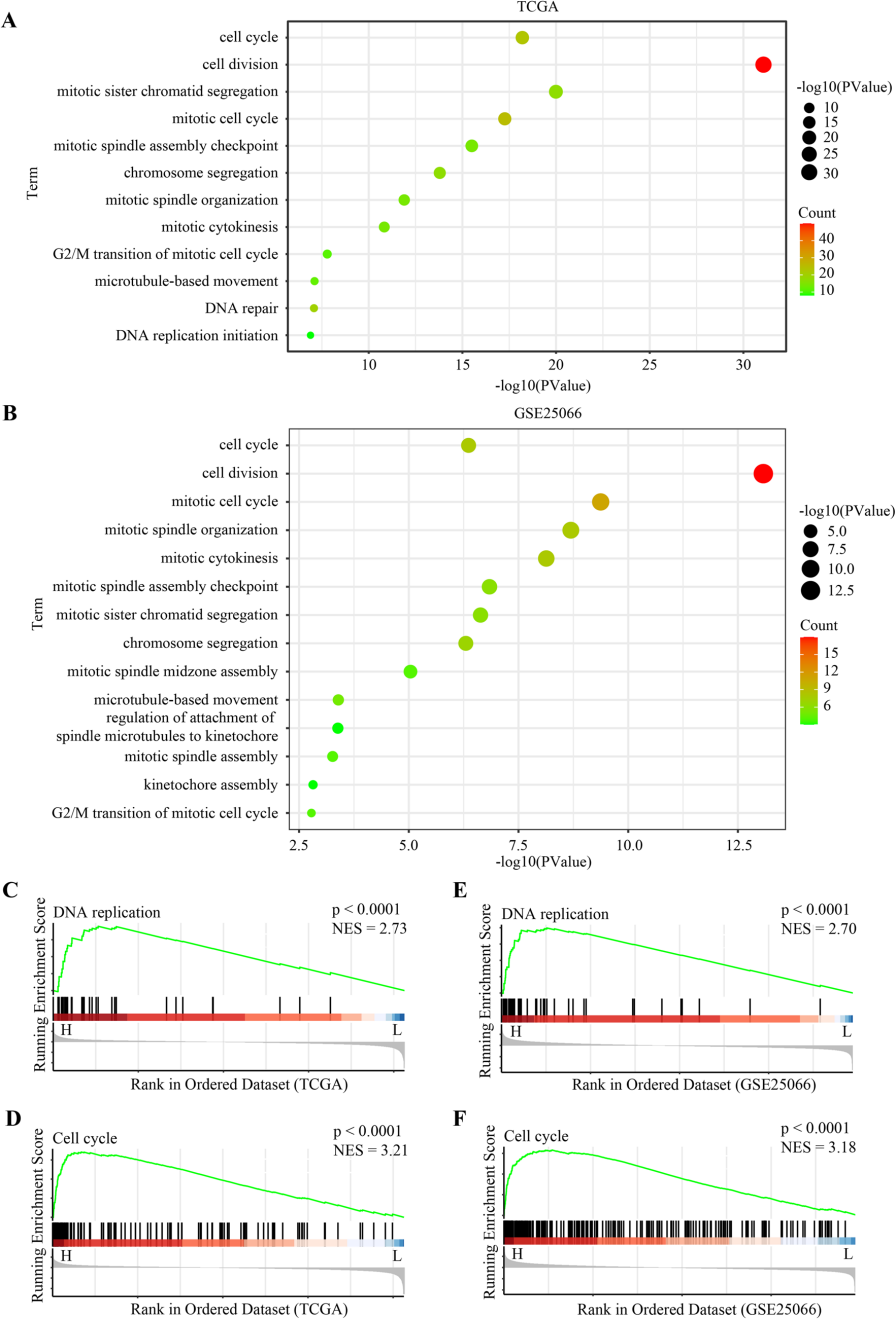
KEGG and GO enrichment analysis using TCGA and GEO databases. **A** Data from TCGA showing enriched pathways or termed in high *ECT2* breast cancers. **B** Data from GSE25066 showing enriched pathways or termed in high *ECT2* breast cancers. **C** GSEA indicating the enrichment of DNA replication pathway in high *ECT2* breast cancers (Based on TCGA). **D** GSEA indicating the enrichment of cell cycle pathway in high *ECT2* breast cancers (Based on TCGA). **E** GSEA indicating the enrichment of DNA replication pathway in high *ECT2* breast cancers (Based on GSE25066). **F** GSEA indicating the enrichment of DNA replication pathway in high *ECT2* breast cancers (Based on GSE25066)

To investigate the biological significance of ECT2 for breast cancer, we performed KEGG and GO enrichment analysis using TCGA and GEO databases. The results showed cell proliferation-associated pathways were significantly enriched in high *ECT2* tumors, including cell cycle, cell division, mitotic sister chromatid segregation, mitotic spindle assembly checkpoint, chromosome segregation, mitotic spindle organization, G2/M transition of mitotic cell cycle, DNA replication initiation, regulation of attachment of spindle microtubules to kinetochore, and kinetochore assembly (Fig. 5A, B). The results of GSEA also demonstrated that DNA proliferation and cell cycle were significantly enriched in high *ECT2* tumors (Fig. 5C–F).

**Fig. 6:**
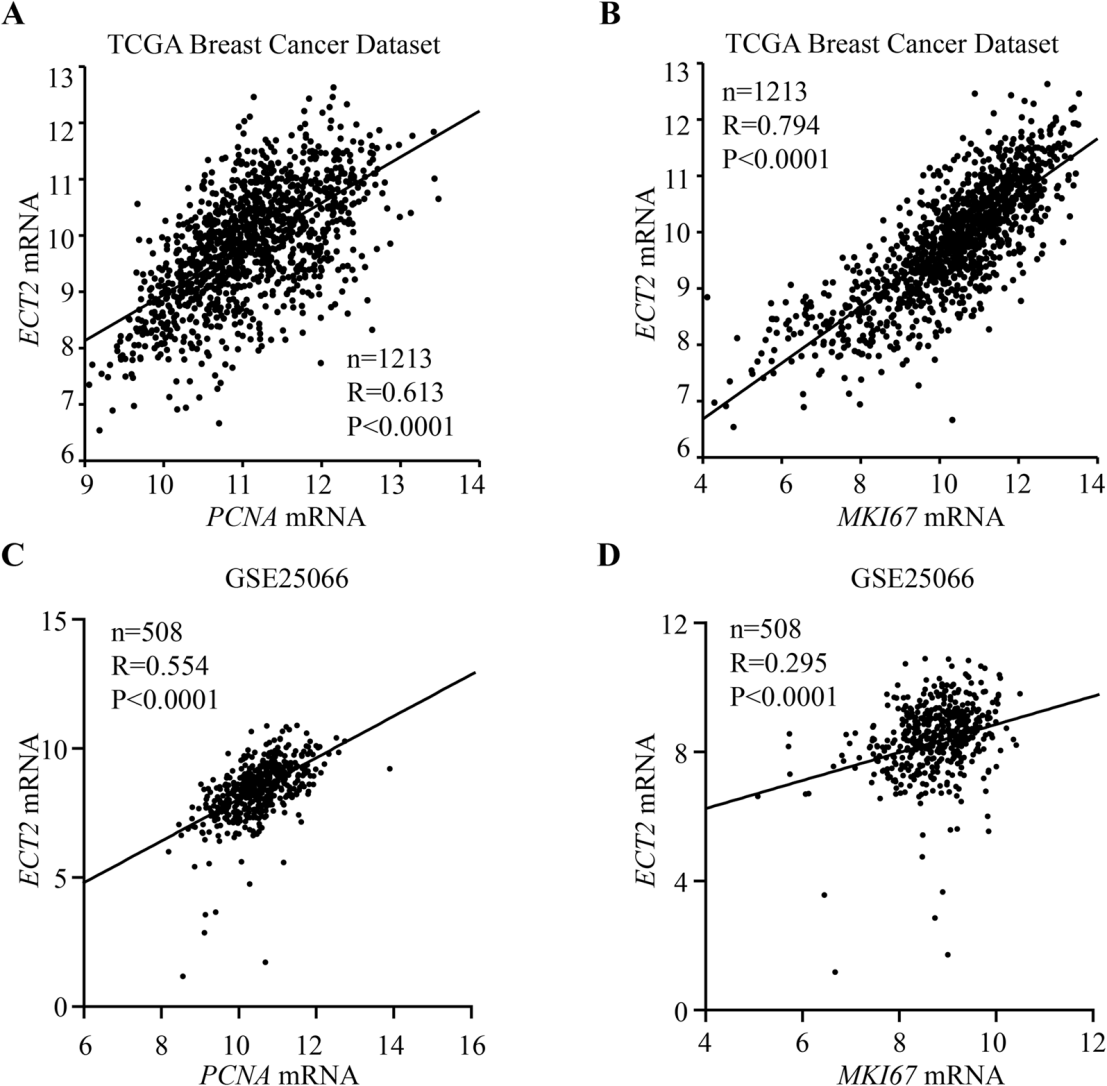
The correlation between *ECT2* expression and cell proliferation-associated markers. **A** Data from TCGA showing correlation between *ECT2* expression and *PCNA* level. **B** Data from TCGA showing correlation between *ECT2* expression and *MKI67* level. **C** Data from GSE25066 showing correlation between *ECT2* expression and *PCNA* level. **D** Data from GSE25066 showing correlation between *ECT2* expression and *MKI67* level

Additionally, as mentioned above, ECT2 plays a vital role in cell proliferation by regulating cytokinesis. PCNA and MKI67 are well-accepted proliferation-associated markers. Therefore, we explored the relationship between ECT2 expression and PCNA and MKI67. We found that *ECT2* expression was highly correlated with cell proliferation-associated markers such as *PCNA* and *MKI67* (Fig. 6A–D). Our results indicated ECT2 participated in cell proliferation, which might contribute to the malignant biological properties of breast cancer.

### Elevated ECT2 expression heralded poor prognosis of breast cancer patients

**Fig. 7:**
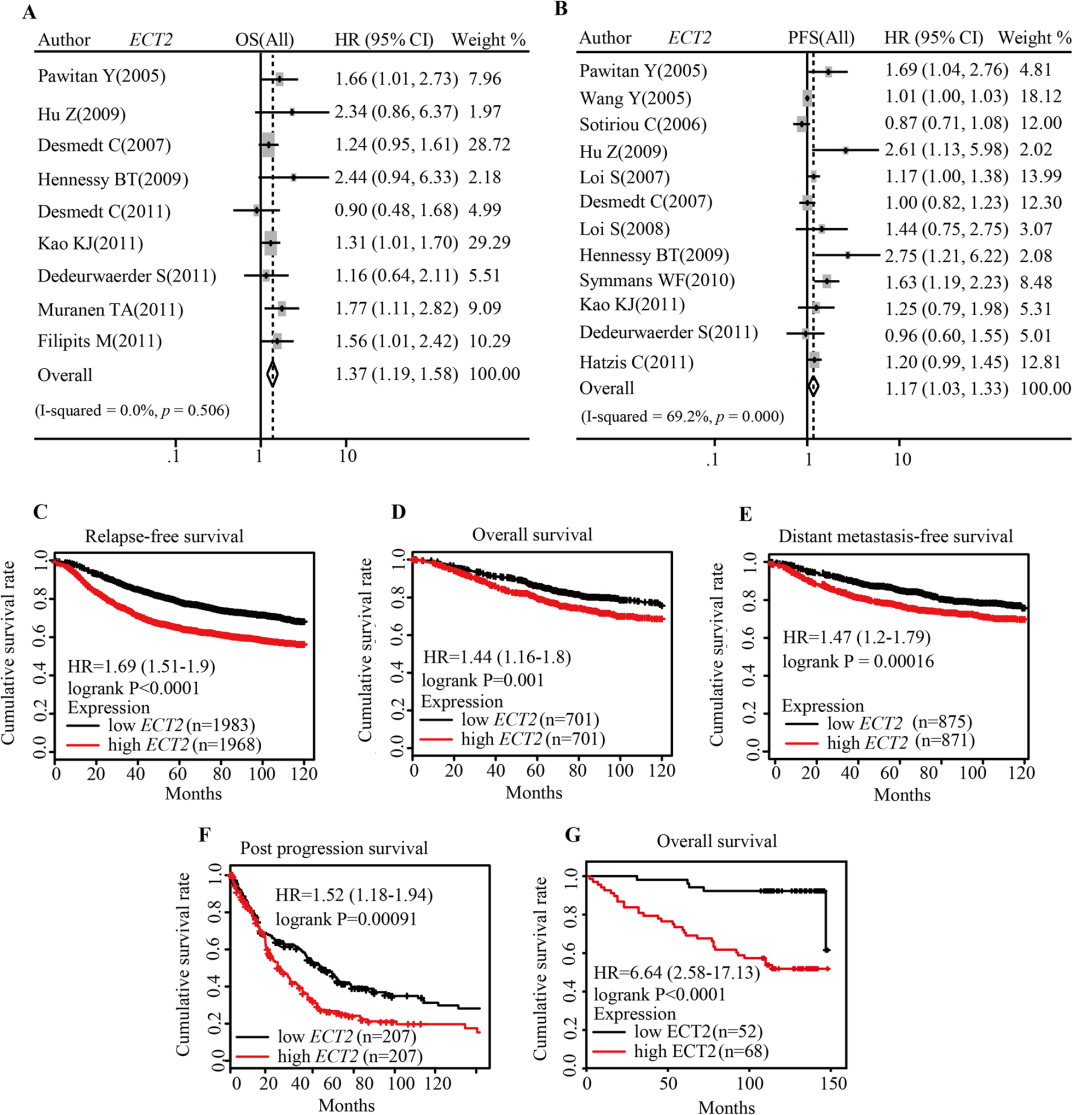
The predictive value of ECT2 for the prognosis of breast cancer patients. **A** Pooled analysis showing the relationship between *ECT2* mRNA level and overall survival. **B** Pooled analysis showing the relationship between *ECT2* mRNA level and progression-free survival. **C** Survival curves showing the relationship between *ECT2* mRNA level and relapse-free survival (data from Kaplan–Meier plotter). **D** Survival curves showing the relationship between *ECT2* mRNA level and overall survival (data from Kaplan–Meier plotter). **E** Survival curves showing the relationship between *ECT2* mRNA level and distant metastasis-free survival (data from Kaplan–Meier plotter). **F** Survival curves showing the relationship between *ECT2* mRNA level and post-progression survival (data from Kaplan–Meier plotter). **G** Survival curves showing the relationship between ECT2 protein level and overall survival (data from tissue microarray)

To assess whether ECT2 expression could predict the prognosis of breast cancer patients, we conducted pooled analysis and found that higher *ECT2* mRNA level related to shorter OS (Pooled HR = 1.37, 95%CI 1.19–1.58) (Fig. 7A) and RFS (Pooled HR = 1.17, 95%CI 1.03–1.33) (Fig. 7B). Survival analysis by online tool Kaplan Meier-plotter showed elevated *ECT2* mRNA level indicated poor prognosis of breast cancer patients (PFS: HR = 1.69, log-rank P < 0.0001; OS: HR = 1.44, log-rank P = 0.001; Distant metastasis-free survival: HR = 1.47, log-rank P = 0.00016; Post progression survival: HR = 1.52, log-rank P = 0.00091) (Fig. 7C–F). Based on patient survival data and ECT2 protein level, survival analysis demonstrated that patients with high ECT2 had shorter OS (HR = 6.64, log-rank P < 0.0001). Cox regression analysis showed that ECT2 expression is an independent prognostic factor for breast cancer patients (Table 2).

**Table 2:** ECT2 expression is an independent prognostic factor for breast cancer patients (HBreD145Su01)

Variables	Univariate analysis		Variable selection	
	HR (95%CI)	*P* Value	HR (95%CI)	*P* value
Age	0.998 (0.970–1.027)	0.895		
Tumor location (left vs. right)	2.077 (0.930–4.638)	0.074		
Grade (grade 2 vs. 1)	1.575 (0.620–3.999)	0.339		
TNM stage (III–IV vs. I–II)	1.979 (0.988–3.966)	0.054		
ER (ER+ vs. ER−)	0.348 (0.157–0.768)	0.009	0.414 (0.185–0.927)	0.032
PR (PR+ vs. PR−)	0.385 (0.173–0.858)	0.020		
Her2 (Her2+ vs. Her2−)	0.906 (0.338–2.428)	0.845		
ECT2 level (high vs. low)	6.198 (2.129–18.043)	0.0001	4.899 (1.668–14.385)	0.004

## Discussion

In this study, by comprehensive analysis of GEO database, TCGA breast cancer dataset, and samples of tissue microarrays, we found that ECT2 expression (at transcriptional and translation levels) was significantly increased in breast cancers compared with non-cancer tissues. Moreover, increased ECT2 expression was related to clinic-pathologic parameters especially advanced TNM stage. We also found ECT2 was remarkably higher in breast cancers belonging to Her2 overexpression and Basal-like subtypes. In general, elevated ECT2 level was a potential biomarker predicting poor prognosis of breast cancer patients. The oncogenic role of ECT2 could be partly fulfilled by enhancing malignant transformation, invasion, and migration activities.

ECT2 is a vital regulator of cell division via modifying the activation of small GTPases of Rho family (e.g. RhoA, Rac1, and Cdc42) [43]. It has been well established that ECT2 activates Rho-Citron kinase pathway which subsequently phosphorylates the myosin heavy chain kinase, induces the formation of contractile ring, and promotes cytokinesis [44]. Apart from cytokinesis, ECT2 also participates in mitosis by impairing the attachment between mitotic spindles and kinetochores [45]. In situ hybridization confirmed that ECT2 level was a biomarker reflecting the proportion of cells undergoing mitosis [46]. In line with the role of ECT2 in cell proliferation, we also found a positive correlation between ECT2 abundance and proliferation-associated biomarkers. Besides, it has been verified that increased ECT2 abundance directly activates the downstream MAPK signaling pathway, further accelerating cell cycle progression and proliferation [33, 47]. Cell proliferation could be effectively inhibited by interfering ECT2 expression [48–50]. Accumulated ECT2 in multiple cancers indicates hyperactive cell division and proliferation [37, 51]. Intervention targeting factors driving the uncontrolled proliferation and disordered cell cycle have always been a hot topic for developing anti-tumor agents [52–55]. Blocking ECT2 and its downstream signaling pathway would be meaningful to transform tumor cells from hyperactive proliferation towards non-division state.

Mis-localized ECT2 relates to malignant transformation, aberrant cell proliferation, and distant metastasis [56]. In cancer tissues, we found abnormal ECT2 staining in cytoplasm. However, in non-cancer tissue, ECT2 staining was only detected in nuclear. It was reported that the splicing variant of ECT2 lacked nuclear localization signal [57]. Even though the oncogenic role of splicing variant of ECT2 without nuclear localization signal was verified in mice model, human cancer cells express full-length ECT2. It is generally believed that transforming ECT2 variant is not related to human cancers. Other factors influencing the subcellular location of ECT2 such as Protein kinase Cι-Par6α complex, are meaningful to counteract malignant behaviors of breast cancers [31, 58].

Besides promoting cell proliferation, the oncogenic role of ECT2 could be attributed to ECT2-Rho pathway-mediated cellular transformation and metastasis [30]. In mice fibroblast models, cytoplasmic ECT2 showed constitutive guanine nucleotide exchange factor activity and effectively induced transformation, while nuclear ECT2 exhibited no malignant transformation activity [28]. In the interphase, due to containing nuclear localization signal, ECT2 is generally separated in nucleus to avoid the activation of downstream Rho GTPases [59]. However, accumulated ECT2 in the cytoplasm of cancer cell leads to hyperactive Rho GTPases, which promotes epithelial-to-mesenchymal transition, loss of cell polarization, formation of invadopodia/ lamellipodia/ filopodia, and tail retraction [24, 30]. As a result, cancer cells undergo malignant transformation with enhanced capabilities of migration, invasion, and metastasis. We found ECT2 expression was markedly elevated in metastatic breast cancer tissues relative to non-metastatic tissues. It has been documented inhibiting the activity of ECT2-Rho pathway could effectively inhibit breast cancer metastasis via modifying actin cytoskeleton remodeling [60]. Given that distant metastasis, together with recurrence, are major causes of breast cancer-related deaths, upregulated ECT2 signaling is a potential target to inhibit the generation of metastatic lesions.

Collectively, increased ECT2 level is highly associated with advanced TNM stage, poor differentiation, and loss of hormone receptors of breast cancer. Integration analysis using GEO public database and tissue microarray indicates that high ECT2 is an adverse prognostic factor for breast cancer patients. We believe the ECT2 level might be a valuable complement for commercially available predictors such as the 21 genes test. Besides, ECT2 would be a novel target for drug development for breast cancer.

## Supplementary Information

**Additional file 1:** **Table S1**: Characteristics of studies involved in meta-analysis.

## Data Availability

The TCGA datasets for this study can be found in the https://xenabrowser.net/datapages/. The GSE25066 datasets for this study can be found in the https://www.ncbi.nlm.nih.gov/geo/query/acc.cgi?acc=gse25066.

## Abbreviations

CI: Confidence interval
ECT2: Epithelial cell transforming 2
ER: Estrogen receptor
GO: Gene ontology
GSEA: Gene set enrichment analysis
Her2: Human epidermal growth factor 2
HR: Hazard ratio
IHC: Immunohistochemical
KEGG: Kyoto Encyclopedia of Genes and Genomes
OR: Odds ratio
OS: Overall survival
MFS: Metastasis-free survival
RFS: Relapse-free survival
PR: Progesterone receptor
