# Supplementary material for: Increased expression of ECT2 predicts the poor prognosis of breast cancer patients
Source: Exp Hematol Oncol. 2022 Dec 26;11:107. doi: 10.1186/s40164-022-00361-3 (PMC9791744; doi:10.1186/s40164-022-00361-3)
Supplement: Supplementary file 1 — Additional file 1: Table S1: Characteristics of studies involved in meta-analysis. [file 40164_2022_361_MOESM1_ESM.docx]

Additional file 1

**Table S1: characteristic of studies involved in meta-analysis**

| **Reference** | **Year** | **Duration(mo.)** | **Grade** | **Patient number** | **Detection** | **Platform** |
| --- | --- | --- | --- | --- | --- | --- |
| Pawitan Y[1] | 2005 | 102 | 1-3 | 159 | Microarray | Affymetrix Human Genome U133A Array |
| Radvanyi L[2] | 2005 | NA | 1-3 | 54 | Microarray | Protein Design Labs Hu03 Custom Affymetrix GeneChip Array |
| Wang Y[3] | 2005 | 180 | NA | 286 | Microarray | Affymetrix Human Genome U133A Array |
| Sotiriou C[4] | 2006 | 210 | 1-3 | 189 | Microarray | Affymetrix Human Genome U133A Array |
| Hu Z[5] | 2009 | 96 | 1-3 | 75 | Microarray | Agilent-012097 Human 1A Microarray (V2) G4110B |
| Richardson AL[6] | 2006 | NA | NA | 40 | Microarray | Affymetrix Human Genome U133 Plus 2.0 Array |
| Ivshina AV[7] | 2006 | 153 | 1-3 | 289 | Microarray | Affymetrix Human Genome U133A Array |
| Minn AJ[8] | 2007 | 156 | NA | 58 | Microarray | Affymetrix Human Genome U133A Array |
| Yu K[9] | 2008 | NA | NA | 270 | Microarray | Affymetrix Human Genome U133A Array |
| Lu X[10] | 2008 | NA | 1-3 | 129 | Microarray | Affymetrix Human Genome U133 Plus 2.0 Array |
| Turashvili G[11] | 2007 | NA | NA | 10 | Microarray | Affymetrix Human Genome U133 Plus 2.0 Array |
| Loi S[12] | 2007 | 177 | 1-3 | 327 | Microarray | Affymetrix Human Genome U133A Array |
| Desmedt C[13] | 2007 | 163 | 1-3 | 198 | Microarray | Affymetrix Human Genome U133A Array |
| Loi S[14] | 2008 | 137 | 1-3 | 77 | Microarray | Affymetrix Human Genome U133 Plus 2.0 Array |
| Hennessy BT[15] | 2009 | 106 | 1-3 | 89 | Microarray | Agilent-012097 Human 1A Microarray (V2) G4110B |
| Schmidt M[16] | 2008 | 240 | 1-3 | 200 | Microarray | Affymetrix Human Genome U133A Array |
| Ma XJ[17] | 2009 | NA | 1-3 | 28 | Microarray | Affymetrix Human X3P Array |
| Desmedt C[18] | 2011 | 182 | 1-3 | 120 | Microarray | Affymetrix Human Genome U133 Plus 2.0 Array |
| Symmans WF[19] | 2010 | 196 | NA | 298 | Microarray | Affymetrix Human Genome U133A Array |
| Sircoulomb F[20] | 2010 | 112 | 1-3 | 51 | Microarray | Affymetrix Human Genome U133 Plus 2.0 Array |
| Tabchy A[21] | 2010 | NA | 1-3 | 178 | Microarray | Affymetrix Human Genome U133A Array |
| Kao KJ[22] | 2011 | 169 | NA | 327 | Microarray | Affymetrix Human Genome U133 Plus 2.0 Array |
| Dedeurwaerder S[23] | 2011 | 109 | 1-3 | 88 | Microarray | Affymetrix Human Genome U133 Plus 2.0 Array |
| Sabatier R[24] | 2011 | 222 | 1-3 | 266 | Microarray | Affymetrix Human Genome U133 Plus 2.0 Array |
| Muranen TA[25] | 2011 | 120 | NA | 183 | Microarray | Illumina HumanHT-12 V3.0 expression beadchip |
| Hatzis C[26] | 2011 | 89 | 1-3 | 508 | Microarray | Affymetrix Human Genome U133A Array |
| Filipits M[27] | 2011 | 211 | NA | 277 | Microarray | Affymetrix Human Genome U133A Array |
| Terunuma A[28] | 2014 | 148 | 1-3 | 61 | Microarray | Affymetrix Human Gene 1.0 ST Array |
| Nagalla S[29] | 2013 | 127 | 1-3 | 139 | Microarray | Affymetrix Human Genome U133A Array |
| Tofigh A[30] | 2014 | 145 | 1-3 | 321 | Microarray | Affymetrix Human Gene 1.0 ST Array |

**NA**, not available;

1. Pawitan Y, Bjohle J, Amler L, Borg AL, Egyhazi S, Hall P*, et al.* Gene expression profiling spares early breast cancer patients from adjuvant therapy: derived and validated in two population-based cohorts. Breast Cancer Res. 2005;7:R953-64.

2. Radvanyi L, Singh-Sandhu D, Gallichan S, Lovitt C, Pedyczak A, Mallo G*, et al.* The gene associated with trichorhinophalangeal syndrome in humans is overexpressed in breast cancer. Proc Natl Acad Sci U S A. 2005;102:11005-10.

3. Wang Y, Klijn JG, Zhang Y, Sieuwerts AM, Look MP, Yang F*, et al.* Gene-expression profiles to predict distant metastasis of lymph-node-negative primary breast cancer. Lancet. 2005;365:671-9.

4. Sotiriou C, Wirapati P, Loi S, Harris A, Fox S, Smeds J*, et al.* Gene expression profiling in breast cancer: understanding the molecular basis of histologic grade to improve prognosis. J Natl Cancer Inst. 2006;98:262-72.

5. Hu Z, Fan C, Livasy C, He X, Oh DS, Ewend MG*, et al.* A compact VEGF signature associated with distant metastases and poor outcomes. BMC Med. 2009;7:9.

6. Richardson AL, Wang ZC, De Nicolo A, Lu X, Brown M, Miron A*, et al.* X chromosomal abnormalities in basal-like human breast cancer. Cancer Cell. 2006;9:121-32.

7. Ivshina AV, George J, Senko O, Mow B, Putti TC, Smeds J*, et al.* Genetic reclassification of histologic grade delineates new clinical subtypes of breast cancer. Cancer Res. 2006;66:10292-301.

8. Minn AJ, Gupta GP, Padua D, Bos P, Nguyen DX, Nuyten D*, et al.* Lung metastasis genes couple breast tumor size and metastatic spread. Proc Natl Acad Sci U S A. 2007;104:6740-5.

9. Yu K, Ganesan K, Tan LK, Laban M, Wu J, Zhao XD*, et al.* A precisely regulated gene expression cassette potently modulates metastasis and survival in multiple solid cancers. PLoS Genet. 2008;4:e1000129.

10. Lu X, Lu X, Wang ZC, Iglehart JD, Zhang X, Richardson AL. Predicting features of breast cancer with gene expression patterns. Breast Cancer Res Treat. 2008;108:191-201.

11. Turashvili G, Bouchal J, Baumforth K, Wei W, Dziechciarkova M, Ehrmann J*, et al.* Novel markers for differentiation of lobular and ductal invasive breast carcinomas by laser microdissection and microarray analysis. BMC Cancer. 2007;7:55.

12. Loi S, Haibe-Kains B, Desmedt C, Lallemand F, Tutt AM, Gillet C*, et al.* Definition of clinically distinct molecular subtypes in estrogen receptor-positive breast carcinomas through genomic grade. J Clin Oncol. 2007;25:1239-46.

13. Desmedt C, Piette F, Loi S, Wang Y, Lallemand F, Haibe-Kains B*, et al.* Strong time dependence of the 76-gene prognostic signature for node-negative breast cancer patients in the TRANSBIG multicenter independent validation series. Clin Cancer Res. 2007;13:3207-14.

14. Loi S, Haibe-Kains B, Desmedt C, Wirapati P, Lallemand F, Tutt AM*, et al.* Predicting prognosis using molecular profiling in estrogen receptor-positive breast cancer treated with tamoxifen. BMC Genomics. 2008;9:239.

15. Hennessy BT, Gonzalez-Angulo AM, Stemke-Hale K, Gilcrease MZ, Krishnamurthy S, Lee JS*, et al.* Characterization of a naturally occurring breast cancer subset enriched in epithelial-to-mesenchymal transition and stem cell characteristics. Cancer Res. 2009;69:4116-24.

16. Schmidt M, Bohm D, von Torne C, Steiner E, Puhl A, Pilch H*, et al.* The humoral immune system has a key prognostic impact in node-negative breast cancer. Cancer Res. 2008;68:5405-13.

17. Ma XJ, Dahiya S, Richardson E, Erlander M, Sgroi DC. Gene expression profiling of the tumor microenvironment during breast cancer progression. Breast Cancer Res. 2009;11:R7.

18. Desmedt C, Di Leo A, de Azambuja E, Larsimont D, Haibe-Kains B, Selleslags J*, et al.* Multifactorial approach to predicting resistance to anthracyclines. J Clin Oncol. 2011;29:1578-86.

19. Symmans WF, Hatzis C, Sotiriou C, Andre F, Peintinger F, Regitnig P*, et al.* Genomic index of sensitivity to endocrine therapy for breast cancer. J Clin Oncol. 2010;28:4111-9.

20. Sircoulomb F, Bekhouche I, Finetti P, Adelaide J, Ben Hamida A, Bonansea J*, et al.* Genome profiling of ERBB2-amplified breast cancers. BMC Cancer. 2010;10:539.

21. Tabchy A, Valero V, Vidaurre T, Lluch A, Gomez H, Martin M*, et al.* Evaluation of a 30-gene paclitaxel, fluorouracil, doxorubicin, and cyclophosphamide chemotherapy response predictor in a multicenter randomized trial in breast cancer. Clin Cancer Res. 2010;16:5351-61.

22. Kao KJ, Chang KM, Hsu HC, Huang AT. Correlation of microarray-based breast cancer molecular subtypes and clinical outcomes: implications for treatment optimization. BMC Cancer. 2011;11:143.

23. Dedeurwaerder S, Desmedt C, Calonne E, Singhal SK, Haibe-Kains B, Defrance M*, et al.* DNA methylation profiling reveals a predominant immune component in breast cancers. EMBO Mol Med. 2011;3:726-41.

24. Sabatier R, Finetti P, Cervera N, Lambaudie E, Esterni B, Mamessier E*, et al.* A gene expression signature identifies two prognostic subgroups of basal breast cancer. Breast Cancer Res Treat. 2011;126:407-20.

25. Muranen TA, Greco D, Fagerholm R, Kilpivaara O, Kampjarvi K, Aittomaki K*, et al.* Breast tumors from CHEK2 1100delC-mutation carriers: genomic landscape and clinical implications. Breast Cancer Res. 2011;13:R90.

26. Hatzis C, Pusztai L, Valero V, Booser DJ, Esserman L, Lluch A*, et al.* A genomic predictor of response and survival following taxane-anthracycline chemotherapy for invasive breast cancer. Jama. 2011;305:1873-81.

27. Filipits M, Rudas M, Jakesz R, Dubsky P, Fitzal F, Singer CF*, et al.* A new molecular predictor of distant recurrence in ER-positive, HER2-negative breast cancer adds independent information to conventional clinical risk factors. Clin Cancer Res. 2011;17:6012-20.

28. Terunuma A, Putluri N, Mishra P, Mathe EA, Dorsey TH, Yi M*, et al.* MYC-driven accumulation of 2-hydroxyglutarate is associated with breast cancer prognosis. J Clin Invest. 2014;124:398-412.

29. Nagalla S, Chou JW, Willingham MC, Ruiz J, Vaughn JP, Dubey P*, et al.* Interactions between immunity, proliferation and molecular subtype in breast cancer prognosis. Genome Biol. 2013;14:R34.

30. Tofigh A, Suderman M, Paquet ER, Livingstone J, Bertos N, Saleh SM*, et al.* The prognostic ease and difficulty of invasive breast carcinoma. Cell Rep. 2014;9:129-42.
